# Surgical outcome in class 4 congenital anomalies of the ossicular chain: a systematic review of the literature

**DOI:** 10.1007/s00405-023-08091-w

**Published:** 2023-07-06

**Authors:** S. E. Henkemans, J. Rovers, H. G. X. M. Thomeer

**Affiliations:** 1 Department of Otorhinolaryngology-Head and Neck Surgery, University Medical Centre Utrecht, 3508 GA Utrecht, The Netherlands; 2 Brain Center, University Medical Centre Utrecht, Utrecht, The Netherlands

**Keywords:** Minor ear anomalies, Otology, Ossicular chain, Audiometry, Malformation, Surgery

## Abstract

**Objective:**

To review hearing and surgical outcomes after reconstructive middle ear surgery in class 4 congenital middle ear anomalies (CMEA), e.g., patients with oval- or round window atresia of dysplasia.

**Data sources:**

Pubmed/Medline, Embase and Cochrane library.

**Review methods:**

Articles containing data on hearing outcomes and complications after reconstructive ear surgery in class 4 anomalies were analyzed and critically appraised. The following data were included and reviewed: patient demographics, audiometric testing, surgical techniques, complications, revision surgeries and their outcomes. Risk of bias was determined, and GRADE certainty of evidence was assessed. Primary outcomes were postoperative air conduction thresholds (AC), change in AC, and success rates (closure of the ABG to within 20 dB), the occurrence of complications (most importantly sensorineural hearing loss) and the long-term stability of hearing results (> 6-month follow-up) and occurrence of recurrence of preoperative hearing loss.

**Results:**

Success rates varied from 12.5 to 75% at long-term follow-up with larger cohorts reporting success rates around 50%, mean postoperative gain in AC varied from 4.7 to 30 dB and − 8.6 to 23.6 dB at, respectively, short- and long-term follow-up. No postoperative change in hearing occurred in 0–33.3% of ears, and recurrence of hearing loss occurred in 0–66.7% of ears. SNHL occurred in a total of seven ears across all studies of which three experienced complete hearing loss.

**Conclusion:**

Reconstructive surgery can be an effective treatment option which should be considered in patients with very favorable baseline parameters, while also considering the substantial risk of recurrence of hearing loss, the possibility of unchanged hearing despite surgery and the rare occurrence of SNHL.

**Level of evidence:**

2c.

**Supplementary Information:**

The online version contains supplementary material available at 10.1007/s00405-023-08091-w.

## Introduction

Congenital middle ear anomalies (CMEA) are a very rare cause of conductive hearing loss (CHL) in children (1–2% of diagnosed middle ear hearing losses) [1]. Usually children with CMEAs present with a concomitant otitis media with effusion (OME), which is the most common cause of hearing loss in 1–6-year-old children. Therefore, initial treatment is usually conservative or by placement of grommets. If CHL persists after OME has been resolved, further assessment and referral by a specialized Tertiary Referral Academic Center might be considered and possibly lead to a CMEA diagnosis. The Cremers and Teunissen classification is most commonly used in the literature regarding CMEAs [2]. In most CMEA cases, ossicular chain reconstruction surgery leads to improved hearing and restores audible communication [3–7]. This is the primary goal of the clinician treating these patients, as hearing deprivation in the young child is detrimental to social, psychological and speech development [8, 9]. Therefore, early treatment is vital, first by audiological support using hearing aids, and possibly reconstructive surgery from the age of 8–10 years. Surgery may not be recommended before this age as the prevalence of recurrent otitis media is high and may lead to disappointing outcomes or complications.

Non-favorable hearing outcomes after surgery occur in the minority of CMEA cases and are more likely in ears with concomitant syndromal diagnoses (around 25% of CMEA patients) and in class 4 anomalies, which are oval- and/or round window anomalies with a possible abnormal course of the facial nerve [10]. In class 4 anomalies, reconstructive surgery consists of creating an opening from middle to inner ear which has been shown to result in more (and more severe) complications compared to class 1–3 anomalies, i.e., considerable recurrence of conductive hearing loss by re-obliteration of the constructed connection to the inner ear and most importantly complete- or partial sensorineural hearing loss (SNHL) [11–13]. To overcome these risks, otologists have suggested opting for audiological hearing amplification instead of surgery. Because this uncertainty around surgery in class 4 CMEA patients remains, it is of value that clinicians are acquainted with the audiometric results and surgical complications reported in the literature. This systematic review provides an overview of the scientific literature in this challenging group of patients and offers evidence for optimal decision-making in treatment strategies.

## Methods

This systematic review was reported according to the PRISMA guidelines. Our protocol was registered in the National Institute for Health Research’s PROSPERO before commencing (Prospero ID: CRD42021229898) [14]. All methodological aspects of this review were carried out independently by two reviewers (SH and JR). Disagreement between reviewers was solved by consensus.

The reviewers searched PubMed/Medline, Embase and Cochrane library, most recently in July 2022. The search terms are available in the supplementary materials.

### In- and exclusion of studies

Studies were selected using the in- and exclusion criteria as defined in the study protocol. Studies were included if: reporting hearing outcome on reconstructive surgery in patients with congenital absence or dysplasia of the oval or round windows, published between 1985 and July 2022 in English, French, Spanish and Dutch. Studies were excluded if surgical methods were not specified or inadequately described, if conducted on animals or cadavers, if less than 5 patients were included or if surgical results were not reported separately from results of non-class 4 CMEA patients.

The reviewers independently selected articles from the search by screening titles and abstracts and analyzed the full texts of all remaining relevant articles.

### Critical appraisal and level of evidence

Assessment of risk of bias and overall study quality was performed using the National Heart, Lung and Blood institute’s Quality Assessment Tool for Before–After (Pre–Post) Studies With No Control Group [15–18]. Criteria 8 ‘Where the people assessing the outcomes blinded to the participants' exposures/interventions?’ and 12 ‘If the intervention was conducted at a group level (e.g., a whole hospital, a community, etc.) did the statistical analysis take into account the use of individual-level data to determine effects at the group level?’ were not used in the critical appraisal as these questions were not applicable to the general literature regarding CMEAs. The studies were classified as good, fair or poor according to pre-specified criteria created to apply to general literature on CMEAs. Studies classified as ‘good’ were considered to be of high methodological quality and have a low risk of bias, ‘fair’ as average quality with moderate risk of bias and poor as low quality and high risk of bias. The certainty of evidence was assessed using Grading of Recommendations, Assessment, Development and Evaluations (GRADE) [19].

### Data extraction

Data were extracted independently by the reviewers using a preset data extraction sheet, disagreement was solved by consensus.

The following data were extracted: study and patient demographics, surgical techniques, results of preoperative and postoperative audiometric testing, i.e., air conduction (AC) pure-tone average (PTA), bone conduction (BC) PTA and air–bone gap (ABG), peri- and postoperative complications, need for revision surgery and findings during revision surgery. For each study, audiometric results were made complete using two out of AC, BC and ABG reported by authors. Audiometric data were extracted in accordance to the committee of hearing and equilibrium guidelines averaging audiometric measures at 0.5, 1, 2, and 3 or 4 kHz. As stated in these guidelines, 3 and 4 kHz were equally accepted and applied accordingly to calculate the PTA [20].

### Outcomes and measures of effect

The primary outcome measures were: postoperative AC and postoperative gain in AC. Audiometric results measured at ≤ 3-month and ≥ 6-month follow-up were used as short- and long-term follow-up. Surgical success was defined by closure of the ABG to within 20 dB. Other definitions of surgical success used in the literature: postoperative AC ≤ 30dB and gain in AC ≥ 15 dB were also extracted if available.

As secondary outcome measures, the incidence of the following complications was defined: postoperative sensorineural hearing loss (SNHL) due to damage to the inner ear during surgery, perilymph gusher, persistent or temporary vertigo/dizziness, postoperative facial nerve weakness, extrusion of prosthesis material and wound infection such as otitis media with or without concomitant labyrinthitis. SNHL was defined as a postoperative decline in BC ≥ 10 dB.

Furthermore, data on stability of hearing results were collected. If reported, results of audiometric testing shortly after surgery (within three months) and at ≥ 6 months were compared. Studies that did not report individual but generic hearing outcome data were also included. Hearing was defined as stable if AC PTA remained within 5 dB of fluctuation from AC PTA measured at short-term follow-up. Other outcome parameters were: (1) need for revision surgery and (2) the incidence of fenestral re-obliteration (diagnosed during revision surgery or on radiologic imaging such as HR-CT).

### Statistical analysis

*P* values < 0.05 were considered as statistically significant. A meta-analysis was not performed as we expected included studies to have a low number of patients due to the rarity of the condition and a high heterogeneity in reported data. Collected data were analyzed in a descriptive manner.

## Results

**Fig. 1:**
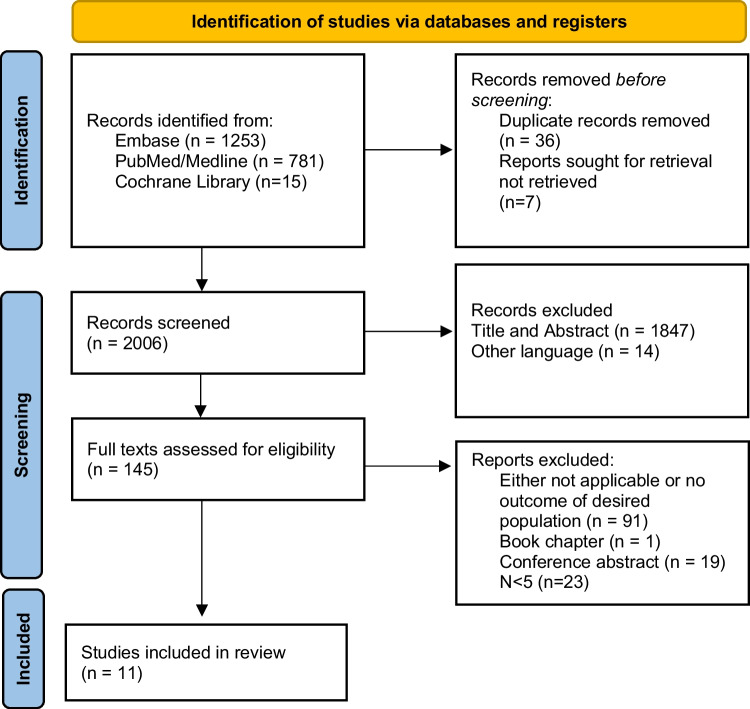
PRISMA flowchart of study selection

11 articles, making a total of 188 operated ears were included in this review. The PRISMA flow chart is displayed in Fig. 1 [11–13, 21–29]. One study was of prospective design, all others were of retrospective design. All studies compared hearing in cohorts or case series of patients pre- and post-surgery without control groups, study characteristics are displayed in Table 1.

**Table 1:** Baseline table of study characteristics

Study	Year	Country	Study design		*N*	Intervention	Primary outcome	FU duration < 3 m	FU duration > 6 m
Ito	2021	Japan	Chart review (MC)	RS	13	NS	Audiometric data	–	1Y
Seidman	2018	USA	Chart review	RS	13	OW drill-out	Audiometric data	Postop^b^	> 1Y
Vincent	2016	France	Case series	PS	11	OW drill-out/stapedotomy	Audiometric data	–	> 1Y 12/15
Su	2014	China	Chart review	RS	56	OW drill-out	Audiometric data	–	6 m
Thomeer	2012	NL	Chart review	RS	8	Neo-OW/stapedotomy	Audiometric data	1	> 1Y
Ashtiani	2010	Iran	Chart review	RS	22	Labyrinthotomy	Audiometric data	–	> 1Y
de Alarcon	2007	USA	Chart review	RS	13^a^	Vestibulotomy	Audiometric data	1	> 6 m
Han	2005	China	NS	–	8	Vestibulotomy	Audiometric data	1	–
Lambert	1990	USA	NS	–	6	Vestibulotomy	Audiometric data	Postop^b^	2.5–5Y
Sterkers	1988	France	NS	–	8	Vestibolotomy	Audiometric data	–	1–18Y
Farrior	1985	USA	NS	–	33	Labyrinthotomy	Audiometric data	Postop^b^	> 2Y (2–30Y)

*FU* follow-up, *MC* multi-center, *NS* not specified, *RS* retrospective, *PS* prospective, *OW* oval window, *Y* year, *M* month

^a^*n* was 13 at short-term FU and 10 at long-term FU

^b^These studies only mentioned that short-term FU was shortly postoperatively without mentioning the duration of short-term FU

### Critical appraisal of risk of bias

Outcome of critical appraisal is displayed in Table 2. One study was classified as good by scoring yes on all criteria within the quality assessment tool. Five studies were classified as fair either by scoring low on how representative the study was, i.e., small patient group and more strict than usual in- and exclusion criteria or scoring low on several criteria. Five studies classified as poor, these all scored moderate to low on how outcome was measured and on other previously stated criteria.

**Table 2:**
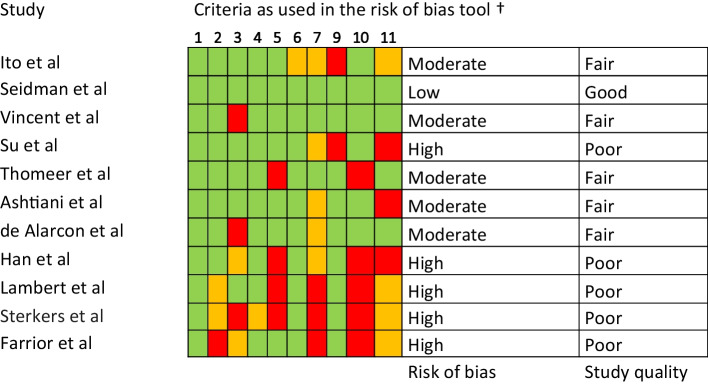
Risk of bias

Green = yes, orange = yes/no, red = no

^†^National Heart, Lung and Blood institute’s Quality Assessment Tool for before–after (pre–post) studies with no control group

### Hearing outcomes

Six studies reported hearing outcomes at ≤ 3 months postoperatively, see Table 3. Mean postoperative AC, reported by three studies, varied from 31.7 dB [13] to 54.6 dB [11]. Mean gain in AC varied from 4.7 dB [11] to 30 dB [13]. The rate of ears that gained AC ≥ 15 dB was reported by three of 11 studies and ranged from 50% (4/8 ears) [11] to 100% (8/8 ears) [29]. The rate of ears that reached postoperative AC thresholds ≤ 30 dB was available in two studies and was 37,5% (3/8 ears) [11] and 78,8% (26/33 ears) [22]. The rate of ears that reached closure of the ABG ≤ 20 dB was available in two studies: 12.5% (1/8 ears [11]) and 61.5% (8/13 ears [24]).

**Table 3:** Audiometric results at short-term follow-up (≤ 3 months)

Study	*n*	AC gain	Postop AC	AC < 30 dB	ABG < 20 dB (*n* (%))	Gain in AC > 15 dB
Seidman	13	21.7	49.6	–	8 (61.5%)	–
Thomeer	8	4.7	54.6	3 (37.5%)	1 (12.5%)	4 (50%)
De alarcon	13	30	31.7	–	–	–
Han	8	–	–	–	–	8 (100%)
Lambert	6	–	–	–	–	4 (66.6%)
Farrior	33	–	–	26 (78.8%)	–	–

Eleven studies reported hearing outcomes at ≥ 6 months postoperatively, see Table 4. Mean postoperative AC, reported by seven studies, varied from 24.6 dB [27] to 52.8 dB [30]. Mean gain in AC varied from – 8.6 dB [31] to 23.6 dB [27]. The rate of ears that gained AC ≥ 15 dB was reported by 6 of 11 studies and ranged from 12.5% (1/8 ears) [11] to 100% (8/8 ears) [29]. The rate of ears that reached postoperative AC thresholds ≤ 30 dB were available in three of eleven studies and ranged from 12.5% (1/8 ears) [11] to 69.7% (23/33 ears) [22]. The rate of ears that reached closure of the ABG ≤ 20 dB was available in five of eleven articles and ranged from 12,5% (1/8 ears [11]) to 75% (6/8 ears [21]).

**Table 4:** Audiometric results at long-term follow-up (≥ 6 months)

Study	*n*	AC gain	ACT	AC < 30 dB	ABG ﻿≤ 20 dB (*n* (%))	Gain in AC > 15 dB	> 5 dB loss of gained AC (*n* (%))	No change AC (*n* (%))
Ito	13	10.5	52.8	–	3 (23%)	–	–	–
Seidman	13	22.7	48.6	–	–	–	0	1 (7.7%)
Vincent	11	22.8	38.4	6 (54.5%)	7 (63.3%)	8 (72.7%)	–	–
Su	56	18.0	49.0	–	29 (51.8%)	29 (51.8%)	–	10 (17.9%)
Thomeer	8	− 8.6	67.9	1 (12.5%)	1 (12.5%)	1 (12.5%)	4 (50%)	–
Ashitiani	22	32.6	24.6	–	–	–	–	–
De alarcon	10^a^	18.4	43.3	–	–	2 (20%)	4 (40%)	–
Han	8	–	–	–	–	8^b^ (100%)	0	–
Lambert	6	–	–	–	–	2 (33.3%)	4 (66.7%)	2 (33.3%)
Sterkers	8	–	–	–	6 (75%)	–	0	1 (12.5%)
Farrior	33	–	–	23 (69.7%)	–	–	2 (6%)	2 (6.1%)

^a^3 patients were excluded because of insufficient follow-up duration

^b^Authors reported stability of hearing results; however, not all patients had follow-up durations ≥ 6 m

Seidman et al., Su et al., Lambert et al., Sterkers et al. and Farrior et al. reported no postoperative change in hearing in, respectively, 7.7% (1/13) of ears, 17.9% (10/56) of ears, 33.3% (2/6) of ears, 12.5% (1/8) of ears and 6.1% (2/33) of ears.

### Stability of hearing results

Six studies reported on the stability of postoperative hearing results. Three studies reported stable hearing results in all ears: Han et al. with a mean follow-up duration of 17.5 months and Seidman et al. and Sterkers et al. both with > 1-year follow-up duration in all ears.

Farrior et al. reported complete loss of gained AC in 6% (2/33) of operated ears within 6 months after surgery and that 69% (23/33) of ears remained at < 30 dB at 2-year follow-up compared to 78.8% (26/33) ears at short-term follow-up (duration not specified). Lambert et al., reported loss of most of the gained AC in 66.7% (4/6) of operated ears during follow-up (> 2 years in all ears). De Alarcon et al. reported loss of gained AC in 40% (4/13 ears), with a mean AC loss of 11.6 dB at > 6 m compared to < 3 m postoperatively. Thomeer et al. reported loss of gained AC in 50% (4/8 ears), with a mean AC loss of 13.3 dB at > 6 m compared to < 3 m postoperatively.

### Complications and revision surgery

Complications are displayed in Table 4. One study (Han et al.) reported no complications occurred. Vertigo was reported by Seidman et al. in 23.1% (3/13) of ears lasting 3 days in two ears and 60 days in one ear which was resolved after revision surgery [24]. Farrior et al. reported mild serous labyrinthitis in 30.3% (10/33) of operated ears which resolved within 2 weeks in all ears. Worsened AC thresholds directly postoperatively or at initial postoperative audiometry were reported by Seidman et al. in 7.7% (1/13 ears) with an 20 dB AC loss and by Thomeer et al. in 37.5% (3/8 ears), with AC losses of 25 dB, 13 dB and 62 dB. Facial paralysis was reported in two studies. De Alarcon et al. reported one case that lasted 4 months; Sterkers et al. also reported facial paralysis in one case that was temporary without mentioning the duration.

SNHL was reported in three articles. Thomeer et al. reported SNHL in 50% (4/8) of ears; one of these ears resulted in total hearing loss after a period of otitis media; in the other ear, BC changed from 20 dB pre-operatively to 80 dB postoperatively without a reasonable explanation; the other two ears experienced BC losses of 14 dB and 18 dB. Seidman et al. reported SNHL (total hearing loss) in one of thirteen ears (7.7%) after revision surgery. Ito et al. reported BC loss > 10 dB in two of thirteen ears (15.4%). Re-obliteration of the oval window was discovered in 6.1% (2/33)–15.4% (2/13) of patients in three different studies. Revision surgery was performed on 16 ears reported by six different studies; findings and outcomes are available in Table 5.

**Table 5:** Complications

Postoperative complications	Ito	Seidman	Vincent	Su	Thomeer	Ashtiani	De Alarcon	Han	Lambert	Sterkers	Farrior
	*n* = 13	*n* = 13	*n* = 11	*N* = 56	*n* = 8	*n* = 22	*n* = 13	*N* = 8	*n* = 6	*n* = 8	*n* = 33
Decrease in AC	–	1 (7.7%)	–	–	3 (37.5%)	–	–	–	–	–	–
Transient vertigo	–	3^a^ (23.1%)	–	–	–	–	–	–	–	–	–
Serous labyrinthitis	–	–	–	–	–	–	–	–	–	–	10 (30.3%)
Facial paralysis^b^	–	–	–	–	–	–	1 (7.7%)	–	–	1 (12.5%)	–
Otitis media	–	–	–	–	1 (12.5%)	–	–	–	–	–	–
Prothesis extrusion	–	–	–	–	–	–	1 (7.7%)	–	–	–	–
Sensorineural Hearing loss	2 (15.4%)	1 (7.7%)	–	–	4 (50%)	–	–	–	–	–	–
Acquired cholesteatoma	–	–	–	–	1 (12.5%)	–	–	–	–	–	–
Small canal inclusion cyst	–	–	–	–	–	–	1 (7.7%)	–	–	–	–
Re-obliteration	–	–	–	–	1 (12.5%)	–	2* (20%)	–	–	–	2 (6.1%)

^a^Duration was 3 days in two ears and 60 days with resolvement after revision surgery in one ear

^b^All studies reported facial paralysis to be < 4 m in duration with complete recovery in all patients

## Discussion

In this systematic review, we reported hearing outcomes of reconstructive surgery in class 4 CMEA patients. A total of 188 ears were included across 11 different studies. Overall, the reviewed data showed promising results. Six of seven studies that reported mean gain in AC ≥ 6 months reported AC gain > 15 dB, four of six studies that reported success rates (closure of the ABG to ≤ 20 dB or AC < 30 dB) ≥ 6 months reported rates > 50%, thereby proving that fenestration surgery can greatly improve hearing in some patients.

However, there also seems to be a varying percentage of ears in which hearing remains unchanged after surgery (in total 16/188 ears, 8.5%) and in which hearing loss recurs after improvement by surgery (in total 14/188 ears, 7.4%). Major permanent complications, i.e., decreased AC and SNHL reportedly occurred in eleven of 188 ears (5.9%) and SNHL specifically in seven of 188 ears (3.7%) ranging from 0 to 50% [11] in individual studies.

Our results negate the belief that surgery in class 4 ears should be avoided entirely, but confirm the belief that fenestration surgery in class 4 ears is less favorable than reconstructive surgery in class 1–3 ears, where success rates vary from 56 to 75% [4, 6, 32].

### Complications and disappointing results

A recurrence of hearing loss and unchanged hearing was reported by several studies in varying percentages. Recurrent hearing loss can be caused by several factors like prosthesis displacement, adhesions in the middle ear, or re-obliteration of the artificially fenestrated area. Surgery is performed to restore the normal ossicular chain conducted acoustic hearing. However, in ears with severe anatomic variations/deformities, as is often the case in syndromal ears, the success rates might be lower [10]. Similar observations are seen in the surgical opening of a congenital bony ear canal atresia (major ear anomalies). In cases with low scores on the Jahrsdoerfer grading scale (more pre-existing anatomical deformities, on a scale from 0 to 10), surgical treatment is not advocated due to its low success rates and high odds of recurrence [33]. Therefore, given the abnormal anatomy in class four CMEA ears, i.e., aberrant facial nerve course, obliterated oval fenestra, it is not surprising that successful outcome is less frequent than in class 1–3 ears and that the middle ear dynamics might be prone to restore the preoperative condition resulting in recurrent fenestral obliteration*.* Unfortunately, the individual causes of complications and disappointing results could not be included in all the cases because of lack of reporting in the included studies.

The most feared complication, complete SNHL or ‘deaf ears’ after surgery occurred in three ears reported by two studies. Partial SNHL occurred in an additional four ears, reported by two studies. Although the incidence of complete SNHL might indeed be low, it is essential to obtain patient informed consent including considering the alternatives, i.e., hearing amplification devices (air and bone conduction hearing aids). Revision surgeries were performed revealing a myriad of different operative findings and varying postoperative results from complete SNHL to great improvement of AC thresholds. Due to disappointing long-term results (see Table 6), surgery could be considered in the minority of cases.

**Table 6:** Revision surgeries, findings and outcomes

Article	Ears revised	Findings	Outcome
Seidman	*n* = 2	Prosthesis too long (1) Evaluation and repositioning of prosthesis (1)	Hearing gain and resolvement of vertigo (1), complete sensorineural hearing loss (1)
Vincent	*n* = 2	TORP inadequate length (1) Piston displacement (1)	Both ABG ≤ 20 dB
Thomeer	*n* = 4	Not reported by authors (1) Postoperative cholesteatoma (1) Obliteration and broken malleus handle (1) Piston displacement (1)	Outcome not provided by authors
De alarcon	*n* = 4	Obliteration (2) Incudal erosion (1) Prosthesis displacement (1)	No hearing gain in all ears, slight worsening in mean BC
Lambert	*n* = 3	Adhesions (2) Wire prosthesis displacement (1)	Hearing did not improve > 10 dB on long-term follow-up in all ears
Sterkers	*n* = 1	Malleus fixation (1)	ABG decreased from 70 to 40 dB

Despite not being able to investigate the effect of a syndromal diagnosis on outcome in class 4 ears due to the lack of data provided in the literature, it is probable that outcome in these ears will be worse than in non-syndromal ears due to non-surgical factors like possible associated inner-ear anomalies as has been shown in syndromal class 1–3 ears [10]. Another factor to consider is recurrent otitis media as middle ear infection is known to be detrimental in operated ears and might result in total SNHL as was the case in one ear reported on by Thomeer et al. [11].

### Alternative treatment modalities

Patients with aforementioned unfavorable factors that might lead to an increased risk of unsuccessful surgery should be counseled for other options such as bone conduction devices (BCD), the vibrant soundbridge and regular hearing aids. Especially in children, alternative treatment modalities (hearing aids) should be considered to start treatment as soon as possible given that surgery is not recommended during the first 6–8 years of life due to the higher prevalence of recurrent otitis media. Other reasons for choosing alternative strategies are ears with an extensive history of middle ear problems (or previous surgeries) as these are at risk for disappointing results due to adhesions and overall rigidity in the ossicular chain and are renowned for their challenging surgical management.

### Decision-making and surgical preparation

It is of importance to note that despite the mentioned risks, surgery in class 4 ears can be of great benefit to the patient by either improving hearing thresholds to functional levels or to make hearing rehabilitation using hearing aids more accessible. Our treatment strategy would be to attempt reconstructive surgery in patients with very favorable baseline parameters, i.e., non-syndromal, no middle ear history and with an accessible operating area (access not completely blocked by the facial nerve and no ear canal atresia). It is, therefore, of utmost importance to obtain a high-resolution computed tomography scanning (HR-CT) of the temporal bone before discussing surgery as an option, to be acquainted with the middle- and inner-ear anatomy for optimal preparation and surgical feasibility. During the decision-making process, patients (and their families) should be informed about the successful long-term hearing outcome rate of around 50%, based on the findings of this review, that some patients might benefit from reconstructive surgery while in other cases surgery might have to be aborted due to unfavorable anatomy and that in some cases hearing is not improved after surgery. Furthermore, patients and family should be informed about the occurrence of re-obliteration, prosthesis misplacement, need for revision surgery and that facial nerve damage or vestibular complaints might happen more frequently than in other CMEAs or in the general population needing middle ear surgery. Lastly, patients should be informed that, although rare, complete hearing loss and postoperative dizziness (vertigo), are a real risk.

### Limitations

The following limitations should be noted: (1) the studies included were heterogenous in patient size, class of surgery conducted and duration of follow-up. Also, audiometric assessment, outcomes and reporting thereof were variable and often incomplete; (2) most of the included studies reported on small patient cohorts or case series; (3) certain important primary and secondary outcome measures reviewed here might be underreported due to vague, or lack of, reporting of individual hearing results in some of the included studies; (4) some studies only mentioned stability of results without providing the most recent hearing outcomes and (5) it was not possible to carry out a meta-analysis due to aforementioned limitations. Lastly, it should be noted that many studies aborted surgery in ears in which the facial nerve completely blocked the original oval window site; therefore, data on surgery in these kinds of ears are very limited.

### Quality of the evidence

We used the GRADE method to assess the certainty of the evidence and strength of recommendation in this review, see Table 7. The evidence regarding all outcomes was of very low certainty which means uncertainty remains of the effects of the intervention, reconstructive surgery, on the outcomes defined in this review. The quality of evidence was influenced by aforementioned limitations, the high to moderate risk of bias identified in all but one study, inconsistency of results in the different studies and imprecision caused by the small sample sizes, incomplete reporting and lack of statistical analysis in most studies.

**Table 7:** GRADE certainty assessment and summary of findings table

Certainty assessment							Summary of findings
Participants (studies) Follow-up	Risk of bias	Inconsistency	Indirectness	Imprecision	Publication bias	Overall certainty of evidence	Effects found
Change in air conduction Thresholds (assessed with: audiometric testing)							
133 (7 observational studies)	Serious^a^	Serious^b^	Not serious	Serious^c^	None	⨁◯◯◯ Very low	Mean gain in AC varied from 4.7 to 30 dB at short-term follow-up (within 3 months postoperatively) Mean gain in AC varied from – 8.6 to 23.6 dB at long-term follow-up (> 6 months postoperatively)
Rate of ears that reached postoperative air–bone gaps ≤ 20 dB (assessed with: audiometric testing)							
129 (6 observational studies)	Serious^a,d,e^	Serious^b^	Not serious	Serious^c^	None	⨁◯◯◯ Very low	Reached ABG ≤ 20 dB ranged from 12.5% (1/8 ears) and 61.5% (8/13 ears) at short-term follow-up (within 3 months postoperatively) Reached ABG ≤ 20 dB ranged from 12.5% (1/8 ears) to 75% (6/8 ears) at long-term follow-up (> 6 months postoperatively)
Rate of ears that reached postoperative air conduction thresholds < 30 dB (assessed with: audiometric testing)							
52 (3 observational studies)	Serious^a^	Serious^g^	Not serious	Serious^c^	None	⨁◯◯◯ Very low	Reached postoperative AC thresholds ≤ 30 dB ranged from 37.5% (3/8 ears) to 78.8% (26/33 ears) at short-term follow-up (within 3 months postoperatively) Reached postoperative AC thresholds ≤ 30 dB ranged from 12.5% (1/8 ears) to 69.7% (23/33 ears) at long-term follow-up (> 6 months postoperatively)
Rate of ears that gained air conduction > 15 dB (assessed with: audiometric testing)							
95 (6 observational studies)	Serious^a,d,e^	Serious^b^	Not serious	Serious^c^	None	⨁◯◯◯ Very low	Gained AC ≥ 15 dB ranged from 50% (4/8 ears) to 100% (8/8 ears) at short-term follow-up (within 3 months postoperatively) Gained AC ≥ 15 dB ranged from 12.5% (1/8 ears) to 100% (8/8 ears) at long-term follow-up (> 6 months postoperatively)
Occurrence of sensorineural hearing loss (postoperative BC decrease > 10 dB) and occurrence of complete SNHL (postoperative BC > 80 dB) (assessed with: audiometric testing)							
188 (11 observational studies)	Serious^a,d,f^	Serious^b^	Not serious	Serious^c^	None	⨁◯◯◯ Very low	SNHL occurred in three studies from 7.7% (1/13 ears) to 50% (4/8 ears), complete SNHL occurred in two studies reporting 7.7% (1/13 ears) and 25% (2/8) ears

^a^Several studies showed moderate to high risk of bias due to inconsistent and incomplete reporting of outcome measures

^b^This outcome measure was variable and, thus, inconsistent across the different studies

^c^Few studies included sufficient patients to provide precise results

^d^Several studies mentioned this outcome without providing individual patient data

^e^Several studies provided mean audiometric outcomes without mentioning this outcome measure

^f^One study provided this outcome measure without providing individual patient data

## Conclusion

Fenestration surgery can be an effective treatment in improving hearing in ears with aplasia or dysplasia of the oval or round window in selected cases. However, the substantial risk of recurrence of hearing loss, the possibility of unchanged hearing after surgery and the rare occurrence of total SNHL should be taken into consideration before opting for surgery.

## Supplementary Information

Below is the link to the electronic supplementary material.

Supplementary file 1 Appendix: Table 1. Search terms (XLSX 11 KB)
